# Association between cancer and allergies

**DOI:** 10.1186/s13223-016-0147-8

**Published:** 2016-08-11

**Authors:** Renata Kozłowska, Andrzej Bożek, Jerzy Jarząb

**Affiliations:** Clinical Department of Internal Disease, Dermatology and Allergology in Zabrze, Medical University of Silesia, Katowice, Poland

**Keywords:** Cancer, Allergy, IgE, Asthma, Allergic rhinitis, Atopic dermatitis

## Abstract

**Background:**

The prevalence of allergies and the incidence of cancer are both increasing worldwide. It has been hypothesized that atopy may affect the risk of some cancers.

**Methods:**

In this study, 1525 patients (754 women and 771 men with a mean age of 52.7 ± 11.9 years) with different types of cancer were examined for the presence of allergies. Allergies were confirmed based on retrospective analysis of allergy diagnostic procedures in patients previously diagnosed with cancer. All patients were also analyzed for bronchial asthma and allergic rhinitis according to relevant guidelines. A control group of patients without cancer diagnoses was used for comparison.

**Results:**

Patients with cancer had significantly fewer IgE-mediated allergic diseases than the control population. For the oncological group compared to the non-cancer patients, the odds ratios (ORs) for allergic rhinitis, atopic dermatitis, and bronchial asthma were 0.67 (95 % CI 0.52–0.81), 0.89 (95 % CI 0.78–0.99), and 1.03 (95 % CI 0.91–1.13), respectively. The mean serum concentrations of total IgE were significantly lower in the study population of patients with cancer than in the patients in the control group (45.98 ± 14.9 vs. 83.2 ± 40.1 IU/l; p < 0.05). There were no significant correlations between the type of cancer diagnosed and the form of allergy.

**Conclusion:**

Our results indicate that the overall incidence of allergies, particularly allergic rhinitis, was lower in patients with some types of cancer. Further studies are needed to confirm our findings.

## Background

The worldwide increased prevalence of cancer has impacted attempts to search for factors that may play roles in promoting or protecting against oncogenesis [1, 2]. Among other approaches, efforts have been made to search for correlations between different chronic diseases, e.g., between allergies and the induction of cancer [3, 4]. The possibility of a promoting or protective role of allergy in cancer has been an interesting research topic over the years. There have been various hypotheses regarding the impact of IgE-mediated allergies and the occurrence of cancer [3]. One hypothesis proposed a protective function for atopies against cancer via immunosurveillance [5–7]. The overstimulation of immunocompetent cells as a result of contact with an allergen causes the production of certain IgE antibodies that may have a cytotoxic influence on cancer cells [5, 7]. A second hypothesis proposed a relationship between atopy and an increased risk of cancer incidence, stemming from the prolonged stimulation of the immunological system by chronic inflammation [5, 7]. A continuous and chronic infection accompanied by an allergy may induce the production of free oxygen radicals, which may encourage oncogenesis [3, 5, 7]. Another hypothesis involves prophylaxis. The physical effects of allergic reactions in some tissues may clear mutagenic triggers before malignant transformations can occur. Finally, another recent hypothesis suggested that inappropriate T-helper 2 immune skewing is possible [8].

Despite a large number of studies, no clear answer regarding the validity of one hypothesis over another has been determined. Retrospective epidemiological observations dominate the literature [6, 9–11]. Most prospective studies have only evaluated self-reported allergy histories and risks of cancer [12, 13]. Only a few studies have estimated allergies in patients with cancer based on the results of an allergy diagnosis (i.e., total IgE measurements and skin prick tests) [14, 15].

The incidence of allergies based on retrospective allergy diagnostic documentation in patients diagnosed with cancer and comparisons with a normal population were explored to attempt to verify one of the aforementioned hypotheses.

## Methods

### Patients

Patients were recruited from eight oncological outpatient clinics in southern Poland. The patient databases from each site were analyzed. A total of 9800 medical records were prescreened, and 3200 patients were randomly selected with the following confirmed diagnoses: breast, lung, colon or skin cancer. The sampled study population was designed to be representative of the most common cancers with respect to typical age and sex in Poland in 2014. From this group, only 1856 patients have to fulfill the following additional inclusion criteria: over 35 years of age and having undergone basic oncological treatment (i.e., chemotherapy, radiotherapy and/or surgery) that was finished a minimum of 1 year prior to the study. From this group of patients, 244 patients were excluded from further analysis due to a lack of detailed documentation, and 87 were excluded due to a lack of consent (exclusion criteria).

In the end, 1525 patients comprising 754 women and 771 men aged 35–80 years were analyzed. This group included 247 patients with lung cancer, 211 patients with colon cancer, 231 patients with breast cancer and 199 patients with skin cancer. The mean time of cancer disease duration was 4.3 ± 2.9 years, and the mean time after basic treatment was 2.7 ± 1.8 years. All types of cancer were previously diagnosed based on clinical symptoms, imaging tests (e.g., X-ray, CT, MRI, PET, endoscopy, and USG), biopsies, serum measurements, cancer markers and the ICD-10 code.

### Control group

A control group was formed from a screening of 10,200 medical records from a medical database of GP in the same region of Poland with an age and sex distribution similar to that of the study group. The control group was randomly selected to maintain similar proportions as those found in the study group. A total of 3100 subjects were randomly selected. The inclusion criteria for the control group were as follows: over 35 years of age and a lack of cancer diagnosis or clinical suspicion.

In total, 1689 subjects were pre-enrolled. The same exclusion criteria were applied as in the study group: lack of detailed documentation (n = 103) and lack of patient consent (n = 42). Finally, 1544 subjects were analyzed. The group comprised 754 women and 790 men, aged 35–82 years.

The population details are presented in Table 1.

**Table 1:** Characteristics of the study population and control group

Features	Study group n = 1525	Control group n = 1544
Percentage of women	49.4	48.8
Mean age (years)	52.7 ± 11.9	50.5 ± 12.4
Smoking-current or past (%)	1115 (73.1)	689 (44.6)
Higher education	368 (24.1)	450 (29.1)
Living in rural area	412 (27.1)	471 (30.5)

### Allergy data

Total serum IgE concentration and specific IgE concentration were analyzed in response to the following allergens: *Dermatophagoides pteronyssinus, D. farinae, Aspergillus fumigatus, Alternaria tennis, Cladosporium herbarum*, dog, cat, grass mix, birch, alder, hazel, and mugwort. IgE values greater than 0.35 kU/l were considered positive.

Skin-prick tests against the mentioned inhalant allergens were also analyzed. The tests were evaluated according to previously published guidelines [16].

Allergic rhinitis was confirmed based on the following medical documentation: laryngological examinations, and history of clinical symptoms according to ARIA guidelines [17]. Bronchial asthma was confirmed by documentation of clinical symptoms according to GINA guidelines and spirometry data and/or positive results from reversibility tests or positive results from methacholine tests in accordance with the criteria of ATS and ERS [18]. Atopic dermatitis was recognized from dermatological examinations and from criteria defined by Hanifin and Rajka [19].

### Statistical analysis

All results are presented as the mean ± SD or as a percentage. Differences between groups were assessed using the Kruskal–Wallis test. The odds ratio was calculated for disease occurrence. A value of p < 0.05 was considered significant for all analyses. Statistica 8.1 was used for the analysis (StatPol, Cracow, Poland).

## Results

**Fig.1:**
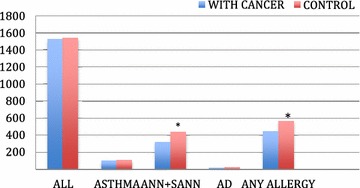
The occurrence of allergic disease in patients with cancer. *ANN* chronic allergic rhinitis*, SAR* sporadic, allergic rhinitis*, AD* atopic dermatitis *p *<* *0.05*

Patients with the analyzed types of cancer had a slightly but significantly lower risk of IgE-mediated allergic diseases than those in the control population. The odds ratio of a clinical manifestation of any allergy (i.e., allergic rhinitis, conjunctivitis, atopic dermatitis, and bronchial asthma) in patients diagnosed with the analyzed types of cancer was 0.76 (95 % CI 0.63–0.84) compared to non-cancer patients. The risk differed depending on the type of allergic disease. The odds ratios for each type of allergy were 0.67 (95 % CI 0.52–0.81) for allergic rhinitis, 0.89 (95 % CI 0.78–0.99) for atopic dermatitis and 1.03 (95 % CI 0.91–1.13) for bronchial asthma. This trend was independent of the type of analyzed cancer with the exception of breast cancer, for which the prevalence of all allergic diseases was the same as for the controls. The details are shown in Fig. 1 and Table 2.

**Table 2:** Percentage of patients with allergic diseases among those with cancer and in the control group

Type of disease	Patients with cancer n = 1525 (%)	Control group n = 1544 (%)	p
Bronchial asthma	5.9	6.1	NS
	4.7^a^		
	6.6^b^		
	7.9^c^		
	4.9^d^		
ANN + SANN	18.9	27.3	0.02
	15.4^a^		
	19.4^b^		
	23.9^c^		
	18.4^d^		
AD	1.9	2.3	NS
	2.4^a^		
	2.8^b^		
	1.7^c^		
	0.9^d^		
Any allergy	24.1	31.2	0.01
	19.1^a^		
	23.4^b^		
	28.9^c^		
	28.1^d^		

*NS* not significant*, AR* chronic allergic rhinitis*, SAR* sporadic, allergic rhinitis*, AD* atopic dermatitis

^a^Lung cancer, ^b^ Colon cancer, ^c^ Breast cancer, ^d^ Skin cancer

There were no significant differences in the allergen profiles of the analyzed allergic patients in the cancer and control groups. However, in both groups, allergies to *D. pteronyssinus* (38.5 % of the study population with cancer vs. 34.2 % of control group), *D. farinae* (31.4 vs. 30.4 %) and grass pollen (24.9 vs. 22.1 %) were predominant.

The mean serum concentration of total IgE was significantly lower in the study population than in the control group (45.98 ± 14.9 vs. 83.2 ± 40.1 IU/l; p < 0.05), and an inverse correlation with cancer incidence was observed. The positive results of analyzed allergen-specific IgEs in allergic patients were comparable between the study and control groups. Based on the skin testing results, IgEs to *D. pteronyssinus, D. farinae* and grass pollen were predominant in both groups.

### Cancer, allergy and socioeconomic factors

Low socioeconomic status (financial problems, low education, smoking, and alcohol use) was related to cancer diagnosis. High levels of education have been linked to allergies. Identical trends of SEP were observed for lung and colon cancer (p < 0.05); however, breast and skin cancer were dependent only on smoking (p < 0.05). There were positive correlations between patient age and diagnosis of all types of cancer (p < 0.05). The influence of socioeconomic factors on the incidence of cancer, allergy or their combination is presented in Table 3.

**Table 3:** Socioeconomic factors (SEP) associated with cancer diagnosis, allergy and their combination—odds ratio in comparison to control patients without diagnosis of cancer and/or allergy

SEP	OR (95 % CI)		
	Cancer n = 1525	Allergy n = 921	Cancer and allergy n = 343
Education			
Low	*1.75 (0.89–1.32)*	0.99 (0.76–1.31)	1.17 (0.98–1.54)
Middle/high	1.23 (0.97–1.76)	*1.87 (0.66–2.03)*	1.34 (1.04–1.83)
Alcohol			
Never	1.37 (0.92–1.77)	0.93 (0.56–1.20)	0.73 (0.34–1.06)
Occasionally	1.28 (0.88–1.96)	1.14 (0.85–1.79)	1.06 (0.78–1.37)
Frequently	*1.94 (1.03–2.43)*	1.09 (0.78–1.53)	1.13 (0.67–1.54)
Smoking			
Never	0.76 (0.32–1.09)	1.06 (0.56–1.34)	0.81 (0.55–1.21)
Past	*1.97 (1.32–3.01)*	1.01 (0.49–1.46)	*1.64 (1.12–2.09)*
Current	*2.09 (1.78–3.54)*	1.27 (0.99–1.96)	*1.97 (1.88–2.87)*
Financial problems			
Yes	*1.66 (1.10–2.13)*	1.31 (0.84–1.81)	1.27 (0.97–1.66)
No	1.41 (1.22–1.73)	1.09 (0.82–1.65)	1.15 (0.69–1.41)
BMI			
<30 kg/m^2^	1.21 (0.78–1.79)	1.13 (0.55–1.63)	1.22 (0.87–1.45)
≥30 kg/m^2^	1.39 (0.89–1.87)	1.05 (0.67–1.32)	1.01 (0.67–1.33)
Sex			
Women	1.11 (0.71–1.38)	1.41 (1.21–1.58)	1.18 (0.82–1.88)
Men	1.28 (0.71–1.94)	*2.08 (1.88–2.73)*	1.41 (0.68–1.91)

All italic values are significant for p < 0.05

*OR* odds ratio*, CI* confidence interval

## Discussion

The results support the hypothesis of a general inverse association between cancer diagnoses and predisposition to allergies. However, this relationship appears to be complex.

The serum concentration of total IgE was inversely correlated with cancer diagnoses. However, there was no correlation between positive results for inhalant allergen-specific IgEs and the overall cancer incidence. Cohort studies have confirmed such a link between total serum IgE and cancer presence. However, these studies analyzed somewhat different types of populations, i.e., patients without initial cancer diagnoses and with only self-reported allergies [11, 14, 20]. Of course, cohorts provide more relevant insights, but case control studies allows an analysis of larger quantities of data (for example, the results of diagnostic procedures) due to their smaller study populations. The present study is also unique in this European region, which is important because of the different life style of the analyzed patients, which influences the prevalence of allergies and cancers.

The protective role of IgE against carcinogenesis was confirmed by Van Hemelrijck et al. [11]. By contrast, Skaaby et al. did not support this hypothesis based on a study of atopy and cancer prevalence in approximately 15,000 patients [21]. Skaaby et al. also noted an association between atopy and a lower risk of dying for breast cancer but not for cancer in general [22]. This study highlighted the lack of a simple link between atopy and the risk of not only neoplasms but also other chronic diseases [22]. These results further support the complexity of this relationship.

We confirmed that sporadic or chronic allergic rhinitis was significantly less prevalent in patients with breast, colon, pulmonary or skin cancer diagnosis. Similar trends between asthma and atopic dermatitis were not determined. It is known that various types of cancers could exhibit different associations with atopic disease. With respect to the prevalence of allergic rhinitis, some studies have confirmed an increase in allergic rhinitis in patients with lung cancer and a lack of association with hematological malignancies [3, 23, 24]. However, as found in the present work, Turner et al. [4, 25] observed a similar reduction in the incidence of intermittent allergic rhinitis in patients diagnosed with colorectal cancer.

The obtained data do not indicate a significant relationship between the analyzed types of cancer and the occurrence of atopic dermatitis. In contrast, an increased or decreased risk of melanoma or non-melanoma cancer in patients with atopic dermatitis was confirmed by other researchers **[**3, 9**].** Despite the high concentrations of IgE in this disease, atopic dermatitis is a more heterogenic disease than allergic rhinitis and also has non-allergic associations (e.g., autoimmunological and infectious diseases).

In a case–controlled study with 3300 incident cancer diagnoses in males from Canada, eczema was found to be less prevalent in patients with cancer [12]. Similar observations were confirmed in a few other studies; however, these studies typically focused on only single types of cancer, e.g., gliomas [13]. Unfortunately these cancer types were not analyzed in the present study.

Bronchial asthma is often considered a disease, particularly with respect to cancer. The patients of the study population with lung cancer did not have a greater incidence of asthma compared with the patients of the control group. However, a few studies have reported that the presence of cancer is either positively or negatively correlated with asthma [3, 12, 26]. Most studies on this relationship have included only small groups of patients and have only explored single types of cancer. The results obtained herein did not examine whether any specific type of inhaled allergy would be more or less favorable in inducing cancer. However, the overproduction of IgE may represent a yet to be determined correlation. The possibility of misclassification of COPD or overlap syndrome (COPD and asthma) during data analysis may also have affected the final results for the odds ratio for asthma in the studied patients.

Additionally, analysis of socioeconomic (SEP) factors confirmed earlier observations that patients with cancer exhibit lower SEP, and the additional coexistence of allergies did not alter this trend in the present study [27]. Higher education and male sex were correlated with the presence of atopic disease, consistent with the observations of a previous study on a Danish population [28]. By contrast, links between atopy and smoking, drinking and BMI were not confirmed by our observations. The atypical nature and relatively small size of the analyzed study group with allergies (>35 years old) could explain these differences. Unfortunately, the applied restrictions for age do not permit an adequate assessment of the relationship between atopy and age. In contrast, the patients with cancer included in this study included a significantly higher number of current or past smokers than those in the control group, which explains their greater propensity toward cancer. This finding have been repeatedly proven [29, 30].

This study was limited by a restricted number of patients and by the analyses of only certain types of cancers. The final number of patients analyzed was also significant reduced due to the exclusion of patients during cancer treatment. However, this likely resulted in more reliable diagnostic results of allergy, particularly IgE, which was measured once. We worried that the concentrations of total and specific IgE in the patient serum during oncological therapy could be underestimated because of its immunosuppressive effect, and this influence cannot be completely excluded. A relationship between IgE and oncological therapy in such patients was not observed.

The assessment of allergies in patients with cancer diagnoses is complicated and affected by a number of factors. Patients and physicians are typically focused on addressing the underlying disease, i.e., cancer. Therefore, in some of the analyzed patients, the diagnosis of allergy was overlooked because of other clinical symptoms.

Furthermore, in addition to skin cancers, we selectively examined the most prevalent types of cancers in the country. Skin cancers were included in our analyses due to the frequency with which skin cancers were observed to coincide with atopic dermatitis, as has been reported in the literature [9, 20, 31]. Our findings confirmed a weak, inverse, and proportional dependence [11].

The influence of oncologic therapies, such as chemotherapy and radiation therapy, could not be excluded from influencing the results of the allergy tests (i.e., false negatives). There were no analyses for IgE-mediated food allergies because of the relative rarity in adults. A small number of positive skin-prick test results and allergen-specific IgEs to foods in the treatment groups resulted in their removal from the final analysis. Finally, the control group was obtained from GP sites, whereas the study patients were obtained from outpatient oncology clinics; although the distributions of age and sex were similar between the two groups, this difference in source may be an important confounder (lack of uniform data, richer medical documentation due to greater attention to patients with cancer).

## Conclusion

The observed association may indicate a dependence between IgE-mediated allergies and reduced prevalence of select types of cancers. In particular, the prevalence of allergic rhinitis was low among oncological study patients. Further studies are needed to confirm our findings.

Additionally, patients with cancer have mostly low socioeconomic status compared to those with allergy.

## References

[1] Dyzmann-Sroka A, Malicki J (2014). Cancer incidence and mortality in the greater Poland region-analysis of the year 2010 and future trends. Rep Pract Oncol Radiother..

[2] Sozańska B, Błaszczyk M, Pearce N, Cullinan P (2014). Atopy and allergic respiratory disease in rural Poland before and after accession to the European union. J Allergy Clin Immunol..

[3] Josephs DH, Spicer JF, Corrigan CJ, Gould HJ, Karagiannis SN (2013). Epidemiological associations of allergy. IgE and cancer. Clin Exp Allergy..

[4] Turner MC (2012). Epidemiology: allergy history, IgE, and cancer. Cancer Immunol Immunother.

[5] Sherman PW, Holland E, Scherman JS (2008). Allergies: their role in cancer prevention. Q Rev Biol..

[6] Heikkilä K, Harris R, Lowe G, Rumley A, Yarnell J, Gallacher J (2009). Associations of circulating C-reactive protein and interleukin-6 with cancer risk: findings from two meta-analysis. Cancer Cause Control..

[7] Siemes C, Visser LE, Coebergh JW, Splinter TA, Witteman JC, Uitterlinden AG (2006). C-reactive protein levels, variation in the C-reactive protein gene, and cancer risk: the Rotterdam study. J Clin Oncol.

[8] Profet M (1991). The function of allergy: immunological defense against toxins. Q Rev Biol..

[9] Arana A, Wentworth CE, Fernández-Vidaurre C, Schlienger RG, Conde E, Arellano FM (2010). Incidence of cancer in the general population and in patients with or without atopic dermatitis in the UK. Br J Dermatol.

[10] Ji J, Shu X, Li X, Sundquist K, Sundquist J, Hemminki K (2009). Cancer risk in hospitalised asthma patients. Br J Cancer.

[11] Van Hemelrijck M, Garmo H, Binda E, Hayday A, Karagiannis SN, Hammar N (2010). Immunoglobulin E and cancer: a meta-analysis and a large Swedish cohort study. Cancer Cause Control..

[12] El-Zein M, Parent ME, Kâ K, Siemiatycki J, St-Pierre Y, Rousseau MC (2010). History of asthma or eczema and cancer risk among men: a population-based case-control study in Montreal, Quebec. Canada. Ann Allergy Asthma Immunol..

[13] Berg-Beckhoff G, Schüz J, Blettner M, Münster E, Schlaefer K, Wahrendorf J (2009). History of allergic disease and epilepsy and risk of glioma and meningioma (INTERPHONE Study Group, Germany). Eur J Epidemiol.

[14] Biggar RJ, Christiansen M, Rostgaard K, Smedby KE, Adami HO, Glimelius B (2009). Immunoglobulin subclass levels in patients with non-hodgkin lymphoma. Int J Cancer.

[15] Eriksson NE, Holmén A, Högstedt B, Mikoczy Z, Hagmar L (1995). A prospective study of cancer incidence in a cohort examined for allergy. Allergy.

[16] Heinzerling LM, Burbach GJ, Edenharter G, Bachert C, Bindslev-Jensen C, Bonini S (2009). GA(2)LEN skin test study I: GA(2)LEN harmonization of skin prick testing: novel sensitization patterns for inhalant allergens in Europe. Allergy.

[17] Bousquet J, Khaltaev N, Cruz AA, Denburg J, Fokkens WJ, Togias A (2008). Allergic rhinitis and its impact on asthma (ARIA) 2008. Allergy.

[18] Global Initiative for Asthma. GINA report, global strategy for asthma management and prevention. 2014. http://www.gina.org. Accessed 23 Aug 2015.

[19] Hanifin JM, Rajka G (1980). Diagnostic features of atopic dermatitis. Acta Derm Venerol Suppl..

[20] Olesen AB, Engholm G, Storm HH, Thestrup-Pedersen K (2005). The risk of cancer among patients previously hospitalized for atopic dermatitis. J Invest Dermatol..

[21] Skaaby T, Husemoen LL, Roswall N, Thuesen BH, Linneberg A (2014). Atopy and development of cancer: a population-based prospective study. J Allergy Clin Immunol Pract..

[22] Skaaby T, Husemoen LL, Thuesen BH, Hammer-Helmich L, Linneberg A (2014). Atopy and cause-specific mortality. Clin Exp Allergy.

[23] Brown LM, Gridley G, Check D, Landgren O (2008). Risk of multiple myeloma and monoclonal gammopathy of undetermined significance among white and black male United States veterans with prior autoimmune, infectious, inflammatory, and allergic disorders. Blood.

[24] Lim WY, Chen Y, Ali SM, Chuah KL, Eng P, Leong SS (2011). Polymorphisms in inflammatory pathway genes, host factors and lung cancer risk in Chinese female never-smokers. Carcinogenesis.

[25] Jacobs EJ, Gapstur SM, Newton CC, Turner MC, Campbell PT (2013). Hay fever and asthma as markers of atopic immune response and risk of colorectal cancer in three large cohort studies. Cancer Epidemiol Biomarkers Prev..

[26] Fan YG, Jiang Y, Chang RS, Yao SX, Jin P, Wang W (2011). Prior lung disease and lung cancer risk in an occupational-based cohort in Yunnan. China. Lung Cancer..

[27] Sommer I, Griebler U, Mahlknecht P, Thaler K, Bouskill K, Gartlehner G (2015). Socioeconomic inequalities in non-communicable diseases and their risk factors: an overview of systematic reviews. BMC Public Health..

[28] Skaaby T, Husemoen LL, Thuesen BH, Jørgensen T, Linneberg A (2015). Lifestyle-related factors and atopy in seven Danish population-based studies from different Time periods. PLoS ONE.

[29] Schaal C, Chellappan SP (2014). Nicotine-mediated cell proliferation and tumor progression in smoking-related cancers. Mol Cancer Res.

[30] Phillips DH (2002). Smoking-related DNA and protein adducts in human tissues. Carcinogenesis.

[31] Ming ME, Levy R, Hoffstad O, Filip J, Abrams BB, Fernández C (2004). The lack of a relationship between atopic dermatitis and nonmelanoma skin cancers. J Am Acad Dermatol.

